# Complete Genome Sequence Analysis of Echovirus 18 Associated with Aseptic Meningitis in Hebei Province, China, in 2015

**DOI:** 10.1128/genomeA.01165-16

**Published:** 2016-10-27

**Authors:** Xiang-peng Chen, Su-zhen Sun, Jia-yun Guo, Jing-jie Li, Zheng-de Xie

**Affiliations:** a Key Laboratory of Major Diseases in Children, Ministry of Education, National Key Discipline of Pediatrics (Capital Medical University), National Clinical Research Center for Respiratory Diseases, Beijing Key Laboratory of Pediatric Respiratory Infection Diseases, Beijing Pediatric Research Institute, Beijing Children’s Hospital, Capital Medical University, Beijing, China; b Children’s Hospital of Hebei Province, Shijiazhuang, Hebei Province, China

## Abstract

Echovirus 18 is a member of the genus *Enterovirus*, family *Picornaviridae*, which can cause meningitis in children. Here, we report the echovirus 18 complete genome sequence, which was isolated from the cerebrospinal fluid of a child with aseptic meningitis in Hebei Province, China.

## GENOME ANNOUNCEMENT

Echovirus 18 (Echo 18) is a member of the genus *Enterovirus*, family *Picornaviridae.* It has been determined to be one of 63 serotypes of the human enterovirus B group, which usually cause mild and self-limited disease and also can cause severe central nervous system infection, such as acute flaccid paralysis (AFP), encephalitis, and aseptic meningitis (1). Echo 18-related neurological diseases have been reported in many countries and regions of the world since it was first discovered in 1955 (2–6). However, to date, only 15 complete genome sequences of Echo 18 are available in GenBank (prototype strain Metcalf, South Korean isolate Kor05-ECV18-054cn, and 13 isolates from Germany submitted in April 2016).

In this study, the Echo 18 strain was isolated from a nine-year-old boy clinically diagnosed with aseptic meningitis in Hebei Province of China in 2015. The patient had acute onset of fever, headache, nausea, and vomiting. A cerebrospinal fluid (CSF) sample was collected within 24 h after the onset of symptoms. The pathogen initially tested positive for enterovirus by real-time reverse transcription-PCR (RT-PCR) and then was further identified as Echo 18 by RT-PCR and sequencing.

The clinical strain was isolated by culturing the CSF in human rhabdomyosarcoma (RD) cell lines. After serial passage and plaque purification, the recovered strain, named E18-314/HB/CHN/2015, was obtained. Viral RNA was extracted with the RNeasy minikit (Qiagen), cDNA was synthesized through reverse-transcribed PCR, and a DNA library was prepared using NEBNext Ultra DNA library prep kit for Illumina, according to the manufacturer’s instructions, and sequenced using the MiSeq platform (Illumina, PE 300). The next-generation sequencing data were assembled using the CAP3 program. Phylogenic analysis was performed by the MEGA6 program.

The complete genome of E18-314/HB/CHN/2015 contains 7,414 nucleotides, including a 5′ untranslated region (5′ UTR) of 743 nucleotides, a 3′ untranslated region (3′ UTR) of 101 nucleotides, and a single open reading frame (ORF) of 6,570 nucleotides encoding a polyprotein of 2,190 amino acids. Comparing the complete genomes of E18-314/HB/CHN/2015 with that of prototype strain Metcalf, the whole-genome nucleotide sequence identity is 80.6%, while the nucleotide sequence identities in the 5′ UTR, P1, P2, P3, 3′ UTR, and VP1 regions are 81.5%, 80.2%, 79.7%, 81.0%, 87.1%, and 80.7%, respectively.

Phylogenetic analysis based on the VP1 sequences of Echo 18 in GenBank showed that all the sequences of Echo 18 isolates from Hebei Province are clustered into a distinct branch.

This report describes the first complete genome sequence of an Echo 18 isolate in China, which belonged to human enterovirus B species in the family *Picornaviridae*.

### Accession number(s).

The complete genomic sequence of E18-314/HB/CHN/2015 was deposited in GenBank under the accession no. KX767786.
